# Concordance analysis of microsatellite instability status between polymerase chain reaction based testing and next generation sequencing for solid tumors

**DOI:** 10.1038/s41598-021-99364-z

**Published:** 2021-10-08

**Authors:** Keitaro Shimozaki, Hideyuki Hayashi, Shigeki Tanishima, Sara Horie, Akihiko Chida, Kai Tsugaru, Kazuhiro Togasaki, Kenta Kawasaki, Eriko Aimono, Kenro Hirata, Hiroshi Nishihara, Takanori Kanai, Yasuo Hamamoto

**Affiliations:** 1 Division of Gastroenterology and Hepatology, Department of Internal Medicine, Keio University School of Medicine, Tokyo, Japan; 2 Keio Cancer Center, Keio University School of Medicine, Tokyo, Japan; 3 Department of Biomedical Informatics Development, Mitsubishi Space Software Co., Ltd., Tokyo, Japan

**Keywords:** Cancer, Genetics, Biomarkers, Oncology

## Abstract

Various malignancies exhibit high microsatellite instability (MSI-H) or mismatch repair deficiency (dMMR). The MSI-IVD kit, a polymerase chain reaction (PCR)-based method, was the first tumor-agnostic companion diagnostic to detect MSI status in MSI-H solid tumors. Recently, next-generation sequencing (NGS), which can also detect MSI-H/dMMR, has been made clinically available; however, its real-world concordance with PCR-based testing of MSI-H/dMMR remains to be investigated. The co-primary end points included the positive and negative predictive values of MSI-H/dMMR. A retrospective analysis of 80 patients who had undergone both MSI testing and NGS between July 2015 and March 2021 was conducted. Five patients were confirmed to have MSI-H in both examinations. Among the 75 patients diagnosed as microsatellite stable (MSS) by PCR-based testing, one with pancreatic cancer was diagnosed as having MSI-H after NGS. One patient with pancreatic cancer was diagnosed as having MSS in both tests was found to have a mutation in *MLH1* by NGS, which was confirmed as dMMR by IHC staining. NGS had positive and negative predictive values of 100% (5/5) and 98.7% (74/75), respectively, for MSI-H. The concordance between NGS and PCR-based testing was 98.8% (79/80). Thus, NGS can be useful for evaluating MSI/MMR status in clinical practice and can be an important alternative method for detecting MSI-H/dMMR in the future.

## Introduction

Recently, immunotherapy has emerged as a new standard of care for patients with various malignancies; however, only few patients have benefited from programmed cell death 1 (PD-1)/programmed cell death ligand 1 (PD-L1) and cytotoxic T lymphocyte antigen 4 (CTLA-4) blockade. High microsatellite instability (MSI-H) and deficient mismatch repair (dMMR) have been among the established biomarkers for predicting response to immunotherapy, regardless of tumor type^1^. Approximately 1%–20% of various advanced malignancies exhibited MSI-H/dMMR, which is associated with specific clinicopathological, genomic, or prognostic features^2–6^. A deficiency in the mismatch repair pathway has been known to induce microsatellite instability (MSI), an accumulation of DNA replication errors, particularly in genome areas with short repetitive nucleotide sequences. dMMR is indicated by the loss of function of MLH1, MSH2, MSH6, or PMS2 proteins, leading to the loss of function of the mismatch repair pathway, which plays a key role in maintaining genomic stability^7^. MSI-H/dMMR tumors produce an increasing number of mutations and neoantigens. CD8^+^ T cells recognize these neoantigens, resulting in immune cell infiltration into tumors higher than microsatellite-stable (MSS) or proficient MMR (pMMR) tumors^8^. Recently, Le et al. reported that patients with MSI-H/dMMR cancers had robust responses to anti-PD-1 antibody^3,9^. Given the promising results, the US Food and Drug Administration (FDA) approved the use of pembrolizumab, a humanized IgG4 monoclonal antibody, for the treatment of patients with unresectable or metastatic MSI-H/dMMR solid tumors^10^.

The MSI-IVD kit (FALCO Biosystems, Kyoto, Japan), which can detect MSI status using a polymerase chain reaction (PCR)-based method, was approved in 2018 as the first tumor-agnostic companion diagnostic for pembrolizumab in patients with MSI-H solid tumors^11^. PCR-based testing is used to determine the MSI status using DNA extracted from tumor tissues without using blood samples as reference and to analyze five mononucleotide repeat markers (BAT25, BAT26, MONO27, NR21, and NR24), which are less susceptible to genetic polymorphisms. The lengths of PCR products from normal DNA are almost confined within the quasimonomorphic variation range (QMVR)^12^. The concordance of PCR-based testing and the standard method using both tumor and normal tissue DNA was evaluated and showed complete consistency^12,13^. Studies have suggested the existence of possible differences in microsatellite marker length according to tumor type. Considering that the PCR-based test is used to evaluate each microsatellite marker on the basis of an QMVR width of ± 3 bases, a slight movement in the wave of each microsatellite marker may result in false-negative results in some solid tumors^14^. Moreover, reports have found differences in the concordance between PCR-based testing and immunohistochemistry (IHC) staining according to tumor types, such as brain tumor, cholangiocarcinoma, ovarian cancer, and endometrial cancer^15–17^.

Recently, next-generation sequencing (NGS) has emerged as an essential tool not only for detecting genomic alterations indicated for molecular targeted agents but also for precise clinical decision making, including risk assessment, diagnosis, and prognosis. Some NGS platforms, irrespective of the type of the commercial or noncommercial base, can also determine MSI status, dMMR, or both. However, differences in the methods used to determine MSI-H have been found across NGS platforms according to the microsatellite markers adopted and algorithm for deciding the MSI-H used. Discrepancies in MSI status assessed using PCR-based testing, NGS, or dMMR by IHC staining have been observed. While approximately 96% concordance between PCR-based testing and IHC staining has been reported, the correlation between PCR-based testing and NGS is yet to be thoroughly investigated^16,18^. Although several studies have reported the concordance between PCR-based testing and NGS and suggested a favorable concordance of 99.4%^19,20^, only few reports have evaluated the real-world concordance of MSI status in PCR-based testing and NGS using data from clinical practice. Given that MSI-H is an established predictive biomarker for immune checkpoint inhibitors, misdiagnosis might influence the therapeutic strategy for patients who would benefit from immune checkpoint blockade. In Europe and the United States, IHC staining has also been recommended for detecting MSI-H/dMMR, which is backed by clinical trials^21^. In Japan, however, PCR-based MSI testing has been the only approved companion diagnostic for pembrolizumab. The present study investigated the concordance between PCR-based testing and NGS and determined the usefulness of NGS for evaluating MSI-H/dMMR using real-world data in Japan.

## Patients and methods

### Patients

Patients with solid tumors who underwent both PCR-based testing and NGS of any platform and were evaluated for their MSI status between July 2015 and May 2020 at Keio University Hospital were retrospectively analyzed. Patients who underwent NGS using the NCC Oncopanel (Sysmex Corporation, Kobe, Japan) were excluded, given that this platform could not evaluate MSI status and alterations in *MSH6* and *PMS2*. Patients with failed analyses of NGS or PCR-based MSI test results due to any reasons were excluded. We obtained information on the patients’ characteristics from their medical records.

### MSI analysis with PCR-based testing

Tumor DNA from deparaffinized cells was analyzed using PCR with five monomorphic mononucleotide repeat markers (BAT25, BAT26, NR-21, NR-24, and MONO-27) developed by the Promega MSI analysis system (Promega Corporation, Madison, WI). Tumor tissue of unstained slides (5–10 pieces of undyed 5-μm specimens) with tumor cells ≥ 50% were required for the analysis. Tumors were classified as MSI-H when two or more of the five markers were positive for shifts in the allelic bands, whereas tumors with one unstable marker were classified as MSI-L and those without any positive marker were classified as MSS^12^.

### NGS-based multiplex gene assay

#### FoundationOne CDx

FoundationOne CDx (F1CDx; Foundation Medicine, Cambridge, MA) was approved for use in all solid tumors in 2019 by the Pharmaceuticals and Medical Devices Agency, a regulatory authority in Japan^22^. Patients with solid tumors who are unresponsive to the standard of care but are eligible for chemotherapy are candidates for this analysis. The test can also be performed in pediatric patients with cancer or patients with orphan cancers as part of the diagnostic process and for developing treatment strategies on the basis of genomic mutation findings. F1CDx can detect substitutions, insertion and deletion alterations, and copy number alterations across 324 genes, selected gene rearrangements, and tumor mutational burden (TMB) using DNA extracted from formalin-fixed paraffin-embedded (FFPE) tumor tissue specimens^23^. To determine MSI status, 95 intronic homopolymer repeat loci (10–20 bp long in the human reference genome) with adequate coverage in the F1CDx assay were analyzed for length variability and compiled into an overall MSI score via principal components analysis^24^. Using the 95 loci for each sample, the repeat length was calculated in each read that spanned the locus, and an MSI score was produced. Each sample was assigned a status of MSI-H or MSS, and samples with low coverage (<  × 250  median) were assigned a status of MSI-unknown (detailed information available at https://www.foundationmedicine.com/genomic-testing/foundation-one-cdx). For the analysis, 10 pieces of undyed 4- to 5-μm sections were prepared from the FFPE specimens, and samples with ≥ 20% tumor content were needed.

#### PleSSision

PleSSision (Mitsubishi Space Software Co., Ltd., Tokyo, Japan), an outsourcing clinical sequencing system, allows for targeted amplicon exome sequencing of 160 cancer-related genes using the Illumina MiSeq sequencing platform (Illumina, San Diego, CA)^25^. Genome annotation and curation for analyzing the sequencing data were performed using an original bioinformatics pipeline called GenomeJack (Mitsubishi Space Software, Tokyo, Japan; http://genomejack.net/english/index.html), in which mapping of the NGS reads to the human reference genome (UCSC human genome 19) was performed using the Burrows–Wheeler Aligner^26^, and the reads were realigned with ABRA^27^. For identification of single-nucleotide variants (SNVs), SAMtools was used to pile up the sequencing reads, and defective SNVs that showed conflict between pairwise reads were abandoned^28^. The criteria for mutations were as follows: the noise distributions arising from random sequencing errors were determined. Each mutation was evaluated using a binomial test (*p* < 0.05) to reduce random sequencing errors. We called somatic mutations by comparing the number of mismatch bases in the tumors with those in the normal controls by using the Fisher exact test (*p* < 0.001). The copy number of each gene was calculated as the median value of all the sequencing reads covering the target genes and compared with the median value of the control samples. In calling copy number alterations (CNAs), we defined more than three-fold copy number increases as “gain” and less than two-fold decreases as “loss.” We identified cancer-specific somatic gene alterations, such as SNVs, insertions/deletions (Indels), and CNAs. Moreover, TMB was measured as a potential biomarker of immunotherapy. In our test, TMB was defined as the number of nonsynonymous and synonymous mutations in the target regions per megabase of tumor genome (the total size of the targeted region in our test was 0.74 Mb), and high TMB was defined as at least 10 mutations per megabase (≥ 10 mut/Mb). All the detected gene alterations in 160 cancer-related genes were annotated and curated using the COSMIC (https://cancer.sanger.ac.uk/cosmic), ClinVar (https://www.ncbi.nlm.nih.gov/clinvar/), CIViC (https://civicdb.org/home), SnpEff^24^, and Clinical Knowledgebase (CKB) databases (https://ckb.jax.org/). This sequencing system evaluated MSI-H/MSS using the MSIsensor program, which reports the percentage of unstable microsatellites as a score^20,29^. In PleSSision, MSIsensor scores ≥ 20 and < 20 are defined as MSI-H and MSS, respectively. For the analysis, 5 pieces of undyed 10-μm sections were prepared from FFPE specimens, and samples with ≥ 20% tumor content were required. Generally, FFPE specimens extracted within 3 years were eligible for evaluation of PCR-based MSI testing and NGS in the present study.

### MMR analysis with IHC staining

The tumor tissues that were categorized as MSI-H by PCR-based testing or NGS were analyzed using IHC. The processed IHC slides were evaluated by two pathologists. Cases with loss of at least one expression of MLH1, MSH2, MSH6, or PMS2 in tumor cells were defined as dMMR. Considering the immunostaining topographic heterogeneity, the IHC results of the MSH2/MSH6 and MLH1/PMS2 patterns were confirmed because MMR proteins function as heterodimers. pMMR was defined as a positive nuclear staining of all MMR proteins.

### Statistical analyses

The positive predictive value of NGS compared with PCR-based testing was calculated as the proportion of the number of patients who were categorized as MSI-H by NGS divided by the number of patients who were considered as MSI-H by PCR-based testing. The negative predictive value of NGS compared with PCR-based testing was calculated as the proportion of the number of patients who were categorized as MSS by NGS divided by the number of patients who were considered as MSI-L/MSS by PCR-based testing. Concordance between NGS and PCR-based testing was calculated as the proportion of the number of patients with MSI-H or MSI-L/MSS categorized by both NGS and PCR-based testing divided by the number of patients. The 95% confidence interval (CI) for the binomial proportion was calculated on the basis of the exact binomial distribution. The frequency and percentage of tumor samples with concordant and discordant MMR statuses based on PCR-based testing and NGS results were calculated, after which the extent of concordance was tested using the Cohen *κ* correlation coefficient (*κ*) with its 95% CI, maximum value *κ* (κmax) given the observed distribution, and exact *p* value. All statistical analyses were performed using the JMP version 14.2.0 software (SAS Institute, Cary, NC).

### Ethical approval statement

This study was approved by the Keio University Hospital Institutional Ethics Committee (approval number: 20200046) and was performed in accordance with the Declaration of Helsinki and Ethical Guidelines for Medical and Health Research Involving Human Subjects. Written informed consent was obtained from all patients.

## Results

### Patient characteristics

**Figure 1:**
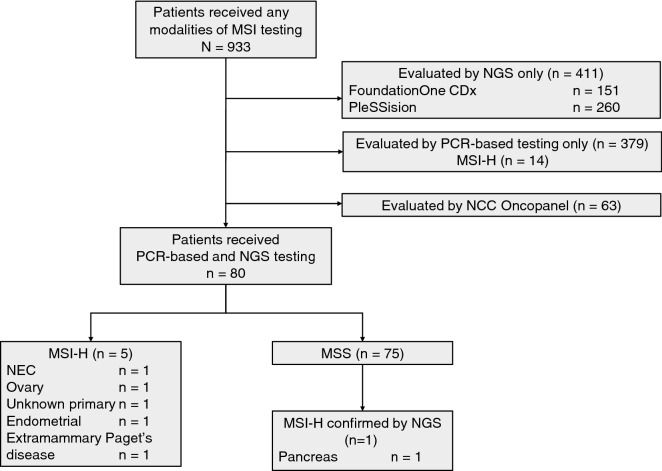
CONSORT diagram of this study. *dMMR* deficient mismatch repair; *MSI-H* microsatellite instability high; *MSS* microsatellite stable; *NEC* neuroendocrine carcinoma; *NGS* next-generation sequencing.

Between July 2015 and March 2021, 933 patients received any modalities of MSI testing, in which 411 and 379 patients underwent NGS or PCR-based MSI testing only, respectively. After excluding 63 patients who were evaluated using the NCC Oncopanel, 80 patients were ultimately included in the present study. A consort flow diagram is presented in Fig. 1. The median age was 62 years (range, 23–89 years), with 36 male patients (45%). Moreover, 58 patients (73%) were evaluated using F1CDx, whereas 22 (27%) were evaluated with PleSSision. The most frequently included tumors were pancreatic ductal carcinoma (n = 13), cervical cancer (n = 10), ovarian cancer (n = 10), extramammary Paget’s disease (n = 9), colorectal cancer (n = 8), and sarcoma (n = 6). The patients’ characteristics are described in Table 1.

**Table 1:** Characteristics of the 80 patients recruited for the present study.

Characteristics	Patients (n = 80)
**Age (years)**	
Median (range)	62 (23–89)
**Sex, n (%)**	
Male	36 (45%)
Female	44 (55%)
**Types of NGS, n (%)**	
FoundationOne CDx	58 (73%)
PleSSision	22 (27%)
**Primary sites, n (%)**	
Pancreas	13 (16%)
Cervix	10 (13%)
Ovary	10 (13%)
Extramammary Paget’s disease	9 (11%)
Colorectal	8 (10%)
Sarcoma	6 (8%)
Endometrial	5 (6%)
Biliary	5 (6%)
Unknown primary	3 (4%)
Central nervous system	3 (4%)
Stomach	2 (3%)
Esophageal	1 (1%)
Head and neck	1 (1%)
Neuroendocrine carcinoma	1 (1%)
Peritoneal	1 (1%)
Thyroid	1 (1%)
Prostate	1 (1%)

*NGS* next-generation sequencing.

### MSI status according to PCR-based testing and NGS

Among the 80 included patients, 5 (5%) with gastric neuroendocrine carcinoma (50-year-old man), ovarian cancer (60-year-old woman), cancer of unknown primary (40-year-old man), endometrial carcinoma (73-year-old woman), and extramammary Paget’s disease (80-year-old man), respectively, were determined as having MSI-H by both PCR-based testing and NGS.

**Figure 2:**
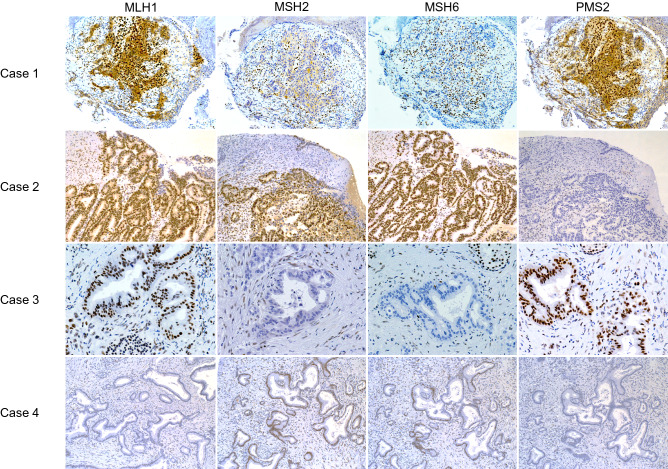
Immunohistochemistry staining of representative cases with MSI-H/dMMR solid tumors.

Of the 75 patients who were initially categorized as having MSI-L/MSS by PCR-based testing, a 47-year-old woman with pancreatic ductal carcinoma was confirmed as having MSI-H and dMMR by NGS (Case 3 in Fig. 2). NGS identified an *MSH2* mutation in the patient, who was also evaluated with IHC staining, which confirmed dMMR (loss of MSH2 and MSH6). She had already been diagnosed with Lynch syndrome and had a family history of cancer.

In case 4, a 78-year-old woman with pancreatic ductal carcinoma was diagnosed as having MSS by both PCR-based testing and NGS. She had a history of Lynch syndrome and a family history of cancer. NGS identified an *MLH1* mutation, and IHC staining confirmed loss of MLH1 and PMS2.

An expert panel discussed the NGS results. Genotype-matched treatments were provided to the five patients, of whom four received pembrolizumab and one received PD-1 inhibitor. Moreover, two partial responses were observed. The characteristics of all seven cases determined to be MSI-H and/or dMMR in this study are described in Table 2.

**Table 2:** Details for the seven cases with deficient mismatch repair (dMMR) and/or high microsatellite instability (MSI-H) confirmed by polymerase chain reaction-based testing and/or next-generation sequencing.

No	Age	Sex	Primary site	Past Medical history	Family histories	PCR	NGS		IHC	TMB (mut/Mb)	Actionable gene alterations*	Use of immune checkpoint blockade, best response
						Result	Platform	Result	Result			
1	50	M	gNEC	None	Primary unknown: aunt	MSI-H	PleSSision	MSI-H	MSH2 and MSH6 loss	52.4	*MSH2* Y408Ffs*5, *GNAS* R844H	Pembrolizumab PR
2	60	F	Ovary	None	Colorectal: mother, Lung: grandfather, Liver: grandfather	MSI-H	PleSSision	MSI-H	PMS2 loss	18.8	*PMS2* R315*, *PIK3CA* C378R, *NF1* Y628Lfs*6, *ARID1A* S617Qfs*2	Pembrolizumab NE
3	47	F	Pancreas	Lynch syndrome: Colorectal; Endometrial; Ovary	Lung: father, uncle; Stomach: uncle; Adrenal gland: grandmother	MSS	PleSSision	MSI-H	MSH2 and MSH6 loss	13.4	*MSH2* T788Nfs*11, *KRAS* Q61R, *GNAS* R844C	Pembrolizumab PR
4	78	F	Pancreas	Lynch syndrome	Pancreas: mother; Stomach: sister, grandmother	MSS	PleSSision	MSS	MLH1 and PMS2 loss	45.6	*MLH1* R385C, *KRAS* G12D	-
5	40	M	Primary unknown	None	Colorectal: father, grandfather; Endometrial: mother	MSI-H	PleSSision	MSI-H			*KRAS* Q61H*, TP53* R175H*, NF2* R57*, ARID2 D196fs*19	PD-1 inhibitor SD
6	73	F	Endometrial	None	Lung: grandfather; Stomach and laryngeal: cousin	MSI-H	FoundationOne CDx	MSI-H		29.0	*AKT1* E40K*, ARID1A* F1999fs*1, *CDKN2A* G67S*, CTNNB1* T31I*, FGFR2* A315T*, MLL2* P467fs*283*, ALL2* Q79fs*51*, MLL2* Q3964*, PIK3CA E542K*, SMAD4* S411fs*17	-
7	80	M	Extramammary Paget’s disease	Prostate	Laryngeal: father	MSI-H	PleSSision	MSI-H		51.0	*MSH2* R383*, ECT2L c.2028 + 1_2018 + 15delinsT*, PTEN* N323fs*, ERRB3* V104M	Pembrolizumab PD

*dMMR* deficient mismatch repair; *F* female; *gNEC* gastric neuroendocrine carcinoma; *IHC* immunohistochemistry; *M* male; *MSI-H* microsatellite instability high; *MSS* microsatellite stable; *NE* not evaluable; *NGS* next-generation sequencing; *PR* partial response; *TMB* tumor mutational burden.

*Gene alterations denote gene mutations and copy number alteration.

### Concordance between PCR-based testing and NGS

For MSI-H detection, the positive and negative predictive values of NGS against PCR-based testing were 5/5 (100%; 95% CI, 65.0–100) and 74/75 (98.7%; 95% CI, 96.3–98.7), respectively. Meanwhile, the concordance rate between PCR-based testing and NGS was 79/80 (98.8%; 95% CI, 94.4–98.8; Table 3). The MSI-H status assessed with NGS or PCR-based testing and dMMR evaluated by IHC were consistent (100%). The 74 patients categorized as MSS by both NGS and PCR-based testing were not evaluated routinely with IHC in this study.

**Table 3:** Concordance and discordance of microsatellite instability status between next-generation sequencing (NGS) and polymerase chain reaction (PCR)-based testing.

NGS assay	PCR-based testing			Immunohistochemistry staining	
	MSI-H (n = 5)	MSI-L/MSS (n = 75)		dMMR, n	pMMR, n
MSI-H (n = 6)	5	1		6	0
MSS (n = 74)	0	74		1*	Not evaluated
Positive predictive value of NGS against PCR-based testing				100% (5/5)	
Negative predictive value of NGS against PCR-based testing				98.7% (74/75)	
Concordance between NGS and PCR-based testing				98.8% (79/80)	

*dMMR* deficient mismatch repair; *MSI-H* microsatellite instability high; *MSI-L* microsatellite instability low; *MSS* microsatellite stable; *NGS* next-generation sequencing; *PCR* polymerase chain reaction; *pMMR* proficient mismatch repair.

*One case was diagnosed as MSS by both PCR-based testing and NGS but was confirmed as dMMR by both NGS and IHC (Case 4 in Table 2).

The tissue samples used for PCR-based testing and NGS were consistent in all the patients. For evaluation of 68% of the patients, tissues obtained from the primary tumor site were used, whereas for 32% biopsied or resected metastatic tissues were examined.

## Discussion

This retrospective study suggests that owing to its sensitivity and specificity, NGS could be considered an alternative to PCR-based MSI testing, the approved companion diagnostic for detecting MSI-H in Japan. To the best of our knowledge, this is the first study to evaluate the real-world concordance and discordance between PCR-based testing and NGS using clinical data in Japan. The six patients included in the study were identified with MSI-H by PCR-based testing or NGS. Among the patients, one (case 3) was initially determined as having MSS by PCR-based testing and subsequently reevaluated as having MSI-H by NGS. Notably, a false-negative result in PCR-based MSI testing was suspected in this case. Generally, reports have shown that the low tumor burden or increase in tumor DNA degradation over time observed in the samples could be attributed to the false-negative results. The PCR-based MSI testing requires at least a tumor content of 50% in tumor samples. Moreover, several studies have reported that Lynch syndrome with germline mutations in *MSH6* or *PMS2* might not necessarily show MSI-H^30,31^. However, the present study used the same samples during PCR-based testing and NGS, with all six patients showing MSI-H in NGS, unlike in the PCR-based testing. Additionally, PCR-based testing used a universal control, whereas certain types of NGS refer to each patient’s peripheral blood sample as control in the evaluation of genomic sequencing, including MSI detection. One case (case 4) was determined as having MSS in both tests; NGS detected an *MLH1* mutation, and IHC staining confirmed dMMR in the patient. MSI status itself is recognized as a surrogate marker of dMMR, whereas the true biomarker for predicting the efficacy of anti-PD-1 antibody is MMR deficiency. Although PCR-based testing could only evaluate MSI status, NGS could analyze both MSI status and gene alterations in MMR genes, which we consider as the strong advantage of NGS. Indeed, NGS permits parallel high-throughput sequencing of a high number of microsatellites and genes and may consequently identify MSI, TMB, and other targetable gene alterations.

The present study included two NGS assays (FoundationOne CDx and PleSSision) in which the detection method of gene alterations is different. FoundationOne CDx evaluates only tumor cells, whereas PleSSision collects peripheral blood mononuclear cells from patients as control. The germline variants are also evaluable in PleSSision, which could help determine mismatch repair gene alterations as pathogenic germline variants. The detection method of MSI in each assay is also different. In FoundationOne CDx, 95 intronic homopolymer repeat loci with adequate coverage in the FoundationOne CDx assay are analyzed for length variability and compiled into an overall MSI score via principal components analysis. PleSSision uses an MSIsensor program for evaluating MSI status, which reports the percentage of unstable microsatellites as a score. Furthermore, the number of targeted genes differs, with 324 genes in FoundationOne CDx and 160 genes in PleSSision, respectively. However, these two NGS assays can evaluate gene alterations in four MMR genes. The detection rate of actionable gene alterations in each NGS assay is 62.9% in F1CDx^32^ and 46% in PleSSision^25^. Although the present study was not aimed at comparing these two assays, we consider that they are equivalent in the evaluation of MSI status and detection of MMR deficiency.

Our study highlights the accuracy of PCR-based MSI testing and the possible usefulness of NGS in clinical practice. Vanderwalde et al. assessed the concordance between PCR-based testing and NGS in 2189 matched cases of 26 cancer types, including 23 cases with MSI-H. In MSI-H detection, NGS had a sensitivity of 95.8% (95% CI, 92.24–98.08), specificity of 99.4% (95% CI, 98.94–99.69), positive predictive value of 94.5% (95% CI, 90.62–97.14), and negative predictive value of 99.2% (95% CI, 98.75–99.57), compared with PCR-based testing^19^. Moreover, Middha et al. validated NGS against PCR-based testing and MMR IHC for 138 colorectal cancers (CRCs), including 24 MSI-H CRCs, and 40 uterine endometrioid cancers (UECs), including 15 MSI-H UECs, and showed a concordance of 99.4%^20^. Although these studies reported concordance between MSI status assessed by NGS and PCR-based testing with a larger number of patients in a laboratory setting, our present study demonstrated the clinical usefulness of NGS to analyze MSI status using real-world data in Japan, where PCR-based MSI testing is the only approved companion diagnostic for the use of immune checkpoint blockade as of now. Furthermore, our study was performed in a situation close to real clinical practice where tissue samples are not abundant or are inadequate for MSI testing analysis, unlike previous reports in which tissue samples were obtained from another study.

NGS can be used to investigate a large variety of gene alterations at one time. Generally, PCR-based MSI testing requires tumor specimens with FFPE block or 5–10 pieces of undyed 5-μm pathological specimens with tumor cells ≥ 50%, which is equivalent or large compared with specimens required for NGS to perform genomic testing, which need 5–10 pieces of undyed 5-μm sections with tumor cells ≥ 20%. The number of tumor samples was either limited, particularly in the patients with pancreatic carcinoma, prostate cancer, or cancer of unknown primary, whose tumor burden is generally low or tumor is difficult to access, such as those obtained through biopsy. Hence, we often experienced problems related to the inability to order new genomic testing kits given the lack of available specimens. However, rebiopsy might be difficult for patients with poor conditions or without superficial metastasis. To address such problems, using NGS initially might be an ideal alternative.

One of the advantages of NGS is its ability to simultaneously detect gene alterations in *MLH1*, *MSH2*, *MSH6*, and *PMS2*^23,25^. Furthermore, certain types of NGS can also analyze germline mutations associated with Lynch syndrome by assessing matched normal-tumor pairs^33^. Although the current NGS assay could not analyze loss of function due to DNA methylation, such an approach could be realized in the near future^34^. Furthermore, by counting thousands of fractional mutations, NGS is able to evaluate TMB. TMB is known to be one of the novel predictive biomarkers for the efficacy of immunotherapy^35,36^. Despite the current lack of approval for the use of pembrolizumab in these populations in Japan, the FDA has approved the use of pembrolizumab for patients with unresectable or metastatic TMB-H (≥ 10 mut/Mb) solid tumors by F1CDx in 2020. Although the cutoff value of TMB-H still remains controversial, clinicians would greatly benefit from knowing TMB values when considering immunotherapy.

Conversely, NGS has some problems that must be considered. First, NGS costs approximately 560,000 yen (5300 US dollars), whereas PCR-based MSI testing costs 20,000 yen (190 US dollars) under the health insurance system in Japan, placing considerable economic burden on the national insurance system. Second, the average turnaround time (TAT) for NGS is approximately 4–5 weeks after sample receipt in the laboratory, which might affect treatment decision making. Furthermore, an expert panel discussion is needed to confirm whether gene alterations are meaningful after results are released.

The usefulness of NGS, including the identification of MSI-H/dMMR status, could supersede that of PCR-based MSI testing, provided that cost reductions and shortening of TAT are addressed. Furthermore, the ability of NGS to be performed earlier and at a more adequate timing has made it an indispensable diagnostic strategy. Moreover, forthcoming advancement would allow MSI-H/dMMR to be evaluated using cell-free DNA from a patient’s blood sample. Accordingly, FoundationOne Liquid CDx (Foundation Medicine, Cambridge, MA) and Guardant360 CDx (Guardant Health, Redwood City, CA), which are types of novel platforms for liquid biopsy, can detect MSI status and were approved by the FDA in 2020^37^. Through liquid biopsy, the MSI/MMR status can be determined more quickly and effortlessly, allowing not only the administration of immunotherapy for patients with MSI-H/dMMR tumors but also the recruitment of patients with certain gene alterations in clinical trials^38^. We believe that NGS should be performed diligently in patients suspected to have MSI-H/dMMR after considering their clinical course, past medical history, and family history, regardless of whether they have been determined as having MSS by PCR-based testing.

Some limitations of the present study inherent to its retrospective nature are as follows: first, this was a single-center study; the sample size was limited with potential bias in patient selection. However, we believe that determining the concordance between NGS and PCR-based MSI testing using data from clinical practice is crucial. Second, we did not usually perform IHC staining in all the cases determined as MSS by PCR-based testing, potentially underestimating the number of patients with dMMR. However, the percentage of MSI-H/dMMR cases in our study was 10%, comparable with those reported in previous reports. Furthermore, the facilities where NGS can be performed are limited, and the conditions for insurance coverage are strictly limited in Japan. Patients with good performance status who have completed the standard treatment or have rare cancers for which a standard treatment has not been established yet are candidates for NGS. Thus, the patients who were included in this study might have been biased, particularly with respect to tumor types. Fourth, the information about the sequencing process of NGS was limited; thus, the success rate of NGS was not calculated in the present study. We aimed to investigate the concordance between the PCR- and NGS-based MSI tests; thus, only the patients who underwent both tests were enrolled in the study. In a previous study, we reported a 99% success rate for CLHURC (the previous version of PleSSision)^25^. Similarly, the sequencing success rate of F1CDx in clinical practice has been reported to be approximately 97%^22^.

In conclusion, NGS has comparable usefulness with PCR-based MSI testing for evaluating MSI/MMR status. Thus, NGS can be expected to become an important alternative method for detecting MSI-H/dMMR in the near future after reducing costs, shortening TAT, and improving accessibility at appropriate testing periods.

## Supplementary Information

Supplementary Information.

## Data Availability

The datasets of the current study are available from the corresponding author on reasonable request.
