# Supplementary material for: Concordance analysis of microsatellite instability status between polymerase chain reaction based testing and next generation sequencing for solid tumors
Source: Sci Rep. 2021 Oct 8;11:20003. doi: 10.1038/s41598-021-99364-z (PMC8501090; doi:10.1038/s41598-021-99364-z)
Supplement: Supplementary file 1 — Supplementary Information. [file 41598_2021_99364_MOESM1_ESM.pptx]

## Slide 1
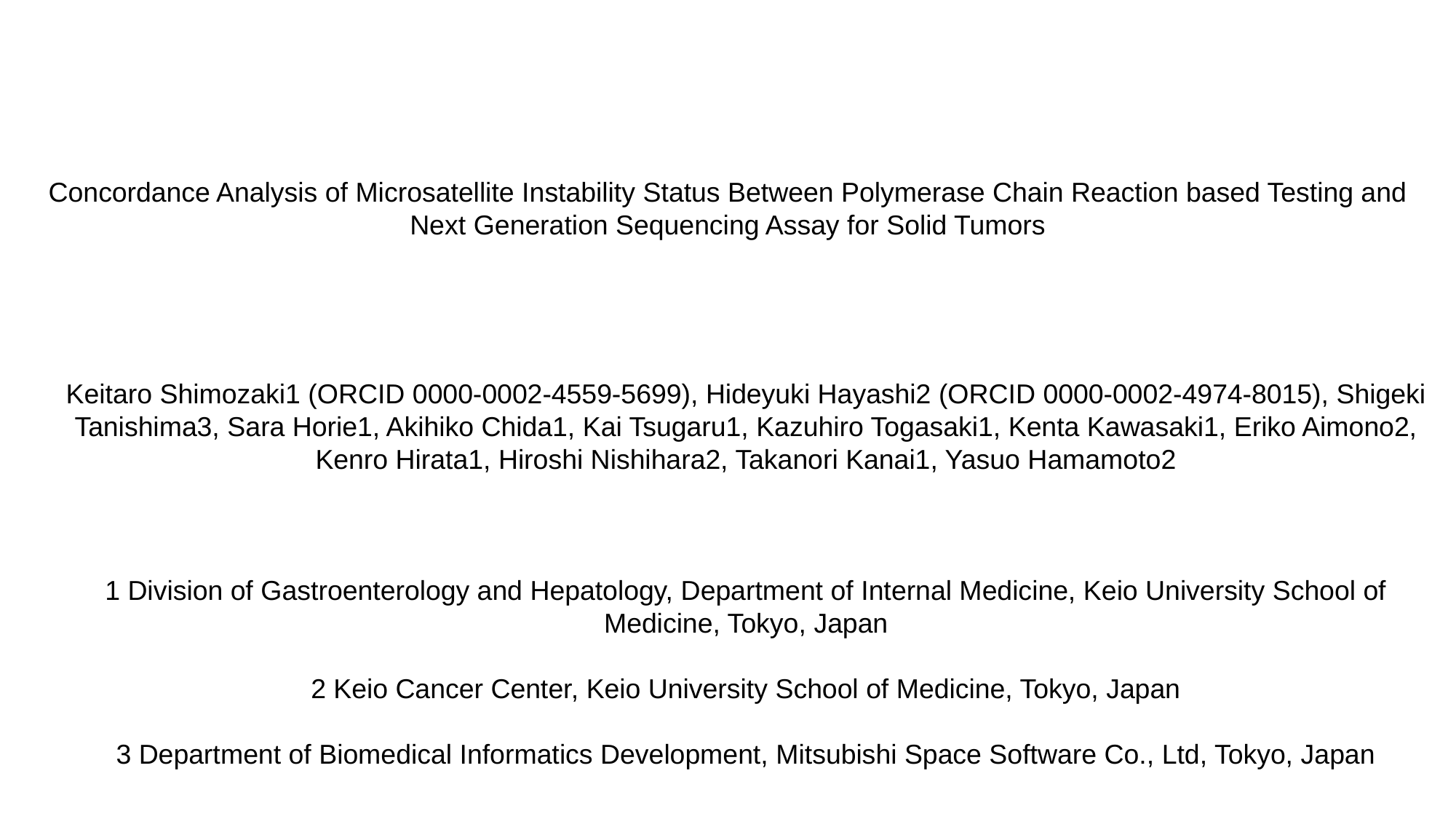

Concordance Analysis of Microsatellite Instability Status Between Polymerase Chain Reaction based Testing and Next Generation Sequencing Assay for Solid Tumors
Keitaro Shimozaki1 (ORCID 0000-0002-4559-5699), Hideyuki Hayashi2 (ORCID 0000-0002-4974-8015), Shigeki Tanishima3, Sara Horie1, Akihiko Chida1, Kai Tsugaru1, Kazuhiro Togasaki1, Kenta Kawasaki1, Eriko Aimono2, Kenro Hirata1, Hiroshi Nishihara2, Takanori Kanai1, Yasuo Hamamoto2
1 Division of Gastroenterology and Hepatology, Department of Internal Medicine, Keio University School of Medicine, Tokyo, Japan
2 Keio Cancer Center, Keio University School of Medicine, Tokyo, Japan
3 Department of Biomedical Informatics Development, Mitsubishi Space Software Co., Ltd, Tokyo, Japan

## Slide 2
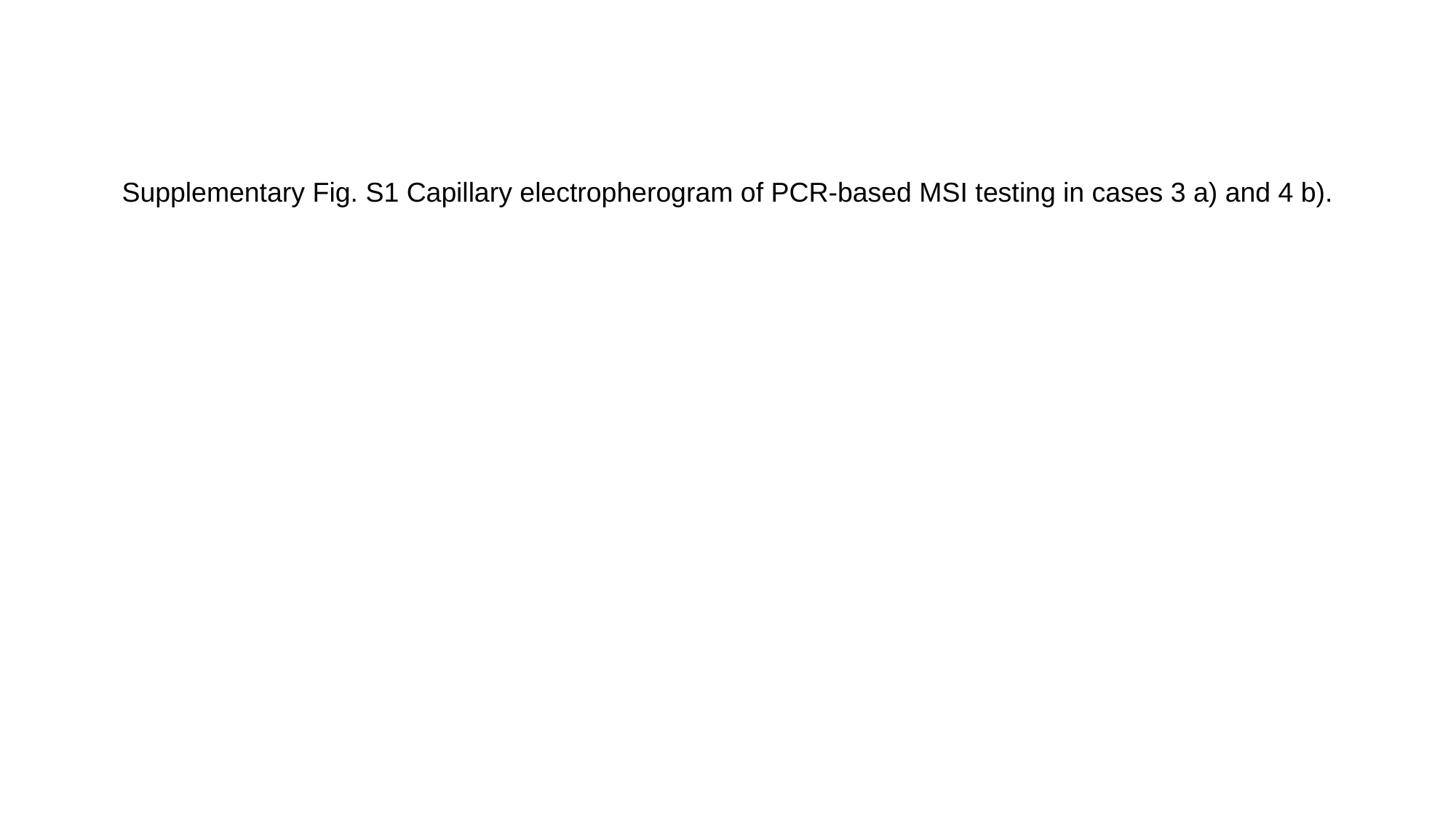

Supplementary Fig. S1 Capillary electropherogram of PCR-based MSI testing in cases 3 a) and 4 b).

## Slide 3
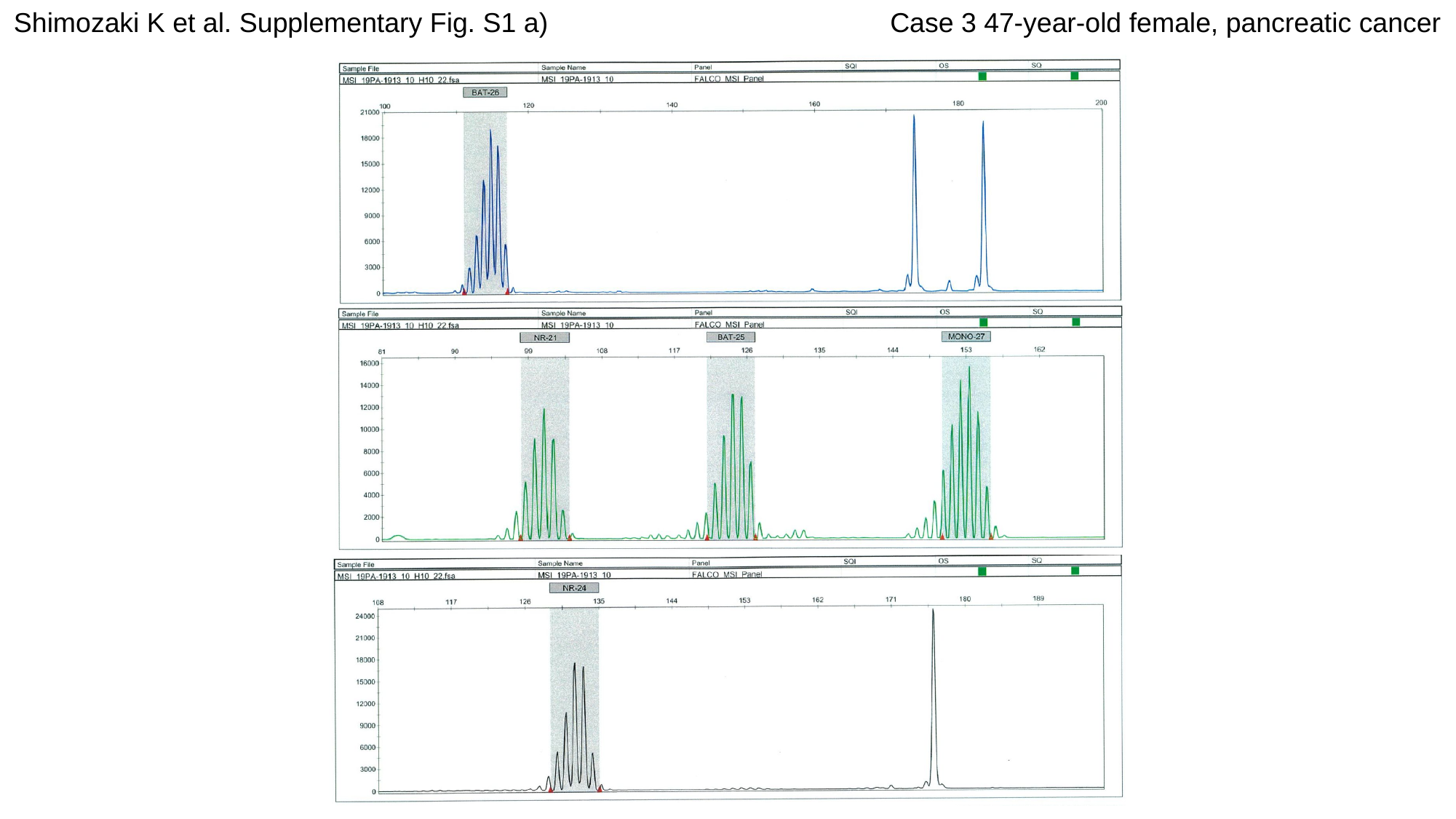

Shimozaki K et al. Supplementary Fig. S1 a)
Case 3 47-year-old female, pancreatic cancer

## Slide 4
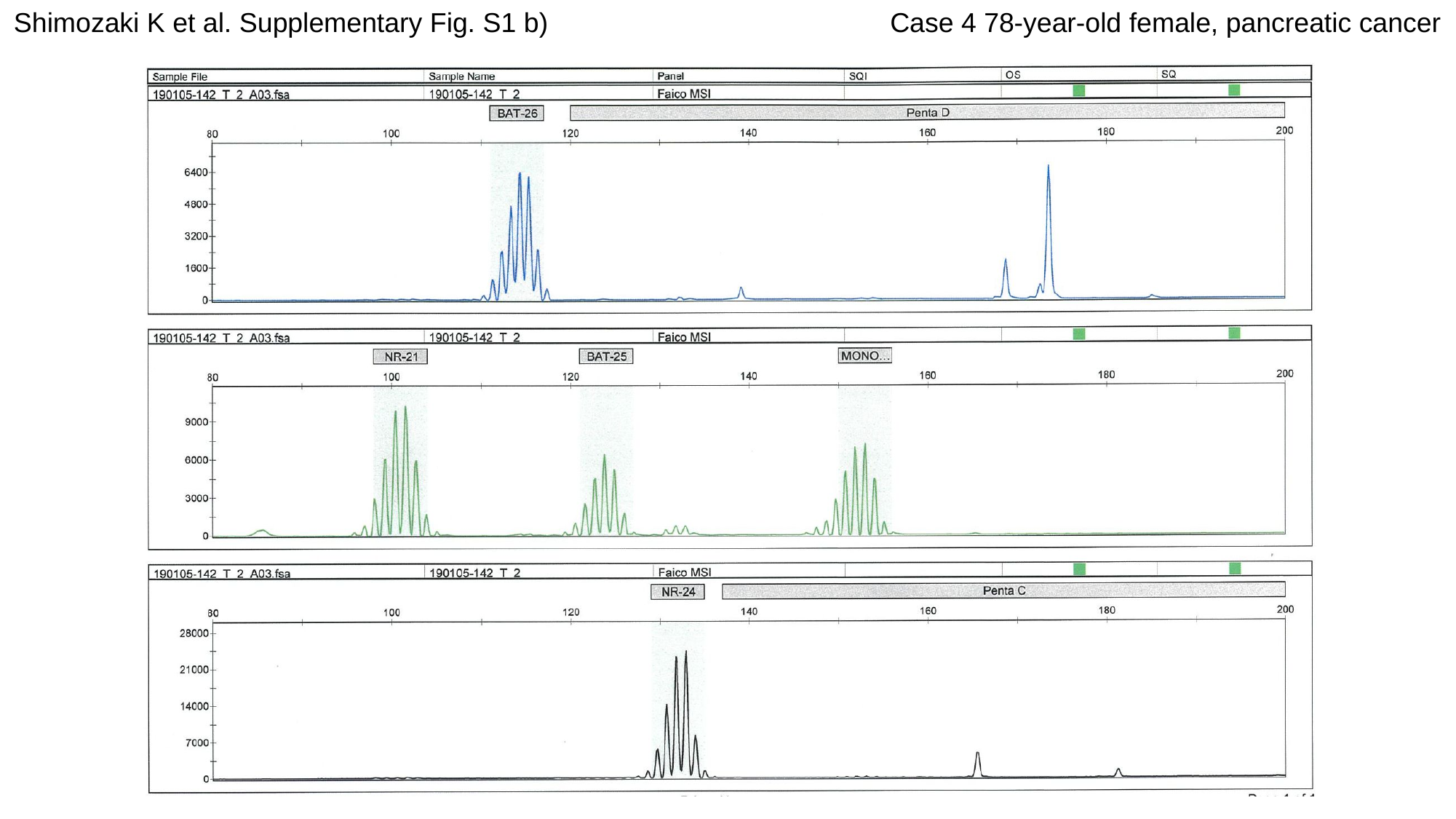

Shimozaki K et al. Supplementary Fig. S1 b)
Case 4 78-year-old female, pancreatic cancer
